# Supplementary material for: The impact of universal recycling on the evolution of economic diversity
Source: PLoS One. 2022 Jan 26;17(1):e0262184. doi: 10.1371/journal.pone.0262184 (PMC8791495; doi:10.1371/journal.pone.0262184)
Supplement: S1 Graphical abstract — (PDF) [file pone.0262184.s001.pdf]

Performance of 30 Pairs of Matched Simulated Model Economies under Two Price-Equilibrium Scenarios

Economic Growth Rate

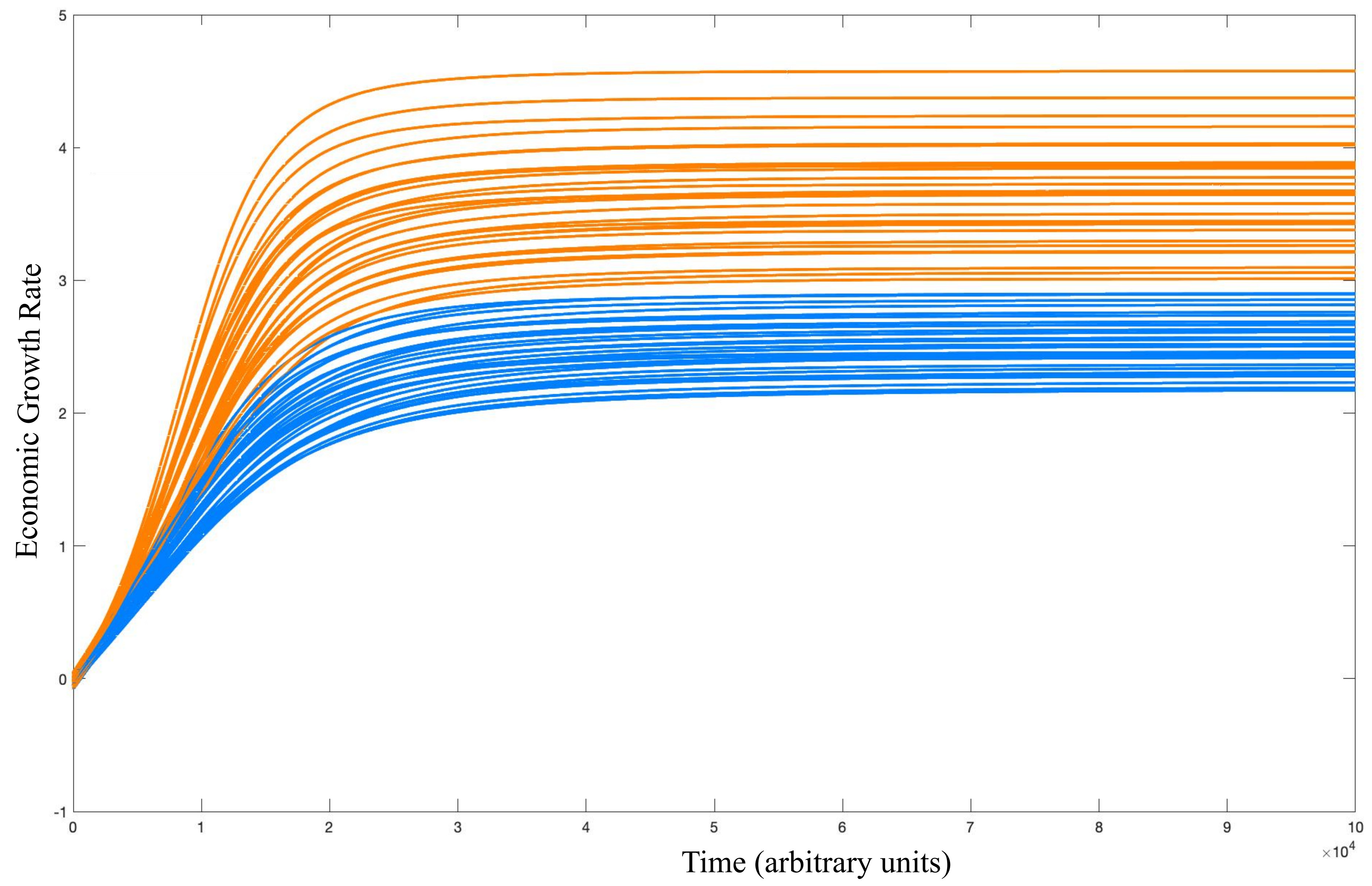

Economic Diversity Index

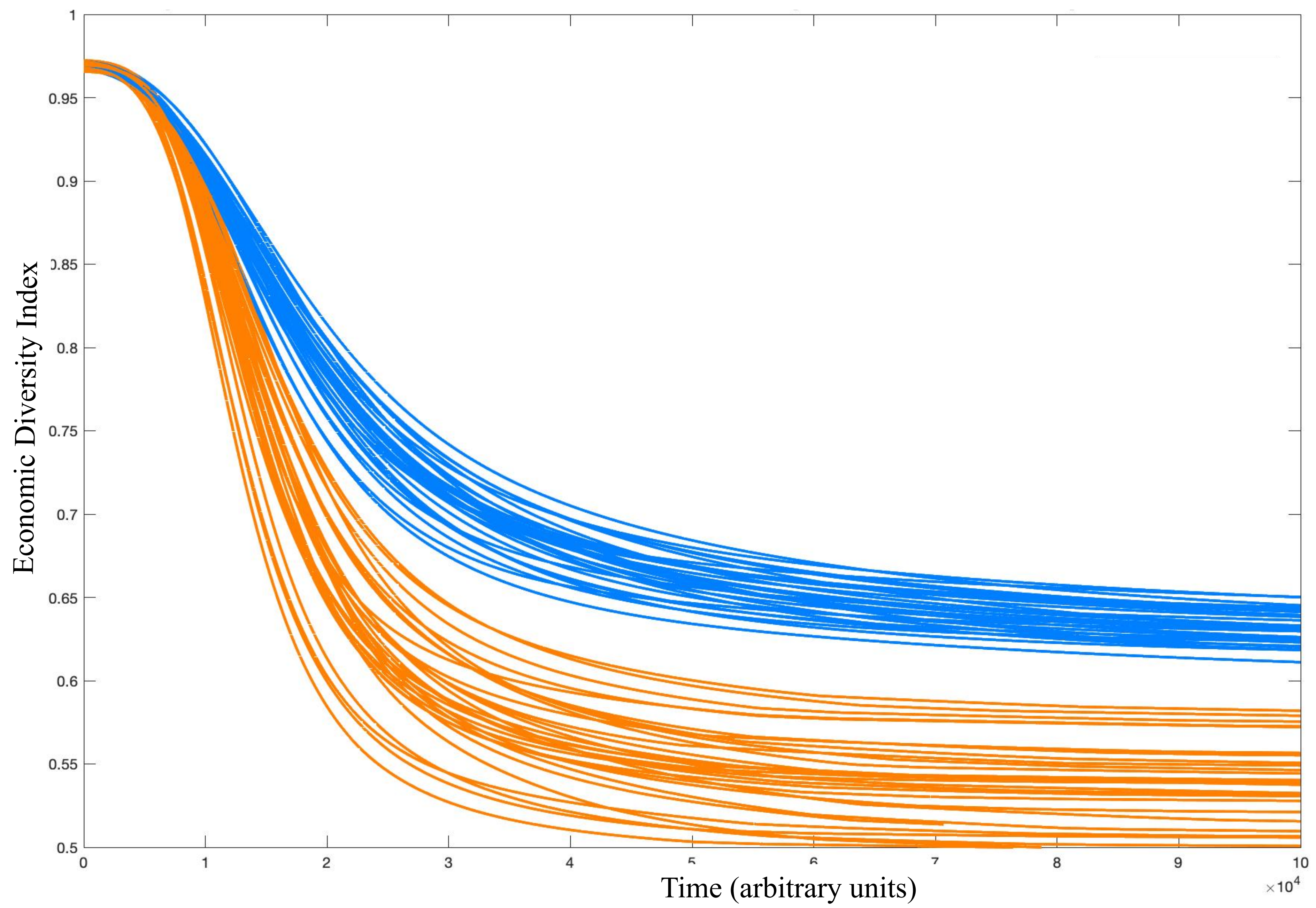

Conventional Price-Equilibrium Scenario

Price-Equilibrium Scenario with Universal Recycling
